# Spring arrival of the common cuckoo at breeding grounds is strongly determined by environmental conditions in tropical Africa

**DOI:** 10.1098/rspb.2023.0580

**Published:** 2023-06-28

**Authors:** Jacob G. Davies, Máire Kirkland, Mark G. R. Miller, James W. Pearce-Higgins, Philip W. Atkinson, Chris M. Hewson

**Affiliations:** ^1^ British Trust for Ornithology Scotland, Stirling University Innovation Park, Beta Centre (Unit 15), Stirling, FK9 4NF, UK; ^2^ British Trust for Ornithology, The Nunnery, Thetford, IP24 2PU, Norfolk, UK; ^3^ School of Biological Sciences, Monash University, Clayton, Victoria 3800, Australia

**Keywords:** common cuckoo, migration timing, ecological constraint, carry-over effects, trade-offs, path analysis

## Abstract

Failure to adapt migration timing to changes in environmental conditions along migration routes and at breeding locations can result in mismatches across trophic levels, as occurs between the brood parasitic common cuckoo *Cuculus canorus* and its hosts. Using satellite tracking data from 87 male cuckoos across 11 years, we evaluate why the cuckoo has not advanced its arrival to the UK. Across years, breeding ground arrival was primarily determined by timing of departure from stopover in West Africa before northward crossing of the Sahara. Together with high population synchrony and low apparent endogenous control of this event, this suggests that a seasonal ecological constraint operating here limits overall variation in breeding grounds arrival, although this event was itself influenced by carry-over from timing of arrival into tropical Africa. Between-year variation within individuals was, in contrast, mostly determined by northward migration through Europe, probably due to weather conditions. We find evidence of increased mortality risk for (a) early birds following migration periods positively impacting breeding grounds arrival, and (b) late birds, possibly suffering energy limitation, after departure from the breeding grounds. These results help identify areas where demands of responding to global change can potentially be alleviated by improving stopover quality.

## 1. Introduction

Climate change has altered the arrival to the breeding grounds of many migratory bird species, with those showing limited responses exhibiting greatest population declines [1]. This may be a consequence of trophic mismatch [2], although the evidence for the expected underlying demographic mechanisms is currently weak [3,4]. In the same way that synchronization of breeding of predators with availability of prey can be disrupted by differential responses to climate change, so too potentially can the synchronization of parasites with optimal periods for exploitation of their hosts. The brood-parasitic common cuckoo *Cuculus canorus* (hereafter ‘cuckoo’) comprises different ‘host races’, individuals specializing in parasitizing different host species. These hosts differ in the timing of breeding and therefore their temporal availability for parasitism by cuckoos. Cuckoo host races have undergone changes in abundance across Europe, apparently due to differential changes in the timing of the long-distance migrant cuckoo's spring arrival to the breeding grounds relative to the breeding of its hosts, which may have contributed to population declines [5,6]. How early the cuckoo arrives on its breeding grounds is therefore biologically important, as well as being of great cultural significance [7].

Tropical-temperate migratory birds generally occupy highly seasonal breeding locations. Consequently, suboptimal timing of arrival can also be highly costly if it is too early [8]. More generally, throughout the annual cycle the timing of migration needs to match the phenology of the successive seasonal locations used, to synchronize individual migrants with resources [9,10]. As processes affecting individual performance may carry-over or correlate between annual cycle stages, it is important to consider the full annual cycle when assessing the causes and consequences of migratory timing [11,12]. Hence, it cannot be ruled out that some population declines attributed to failure to advance arrival to the breeding grounds [1] may be due to other factors correlated with lack of breeding advancement [3], such as constraints on migratory performance earlier in the annual cycle [13]. Currently, our understanding of which events in the annual cycle of migratory birds are most important in determining fitness and how they interact is limited to specific cases in larger species [14,15], primarily due to the difficulty of following individual birds throughout their annual cycle [12].

Restricted advance of breeding grounds arrival in migratory species may be due to three broad non-mutually exclusive factors: (i) limited requirement for earlier arrival, due to, for instance, ability to mitigate the phenological advance of lower trophic levels via dietary flexibility or by shortening the period between arrival and breeding; (ii) limited net selection for earlier arrival, where the survival costs outweigh the reproductive benefits, potentially due to adverse and possibly unpredictable conditions on migration or early in the breeding season [8,16]; and (iii) constraints on response to selection for earlier breeding grounds arrival, if environmental conditions inhibit the ability of birds to advance their migration schedule [17,18]. The latter is possible if responses are inhibited by mechanisms determining migration timing at the individual level [19] or by the information content of cues used to time onward migration to distant locations [20].

Previous studies showing shifts in the host race composition of cuckoos in Europe in relation to climate change, specifically a shift towards use of later breeding long-distance migrants [5,6], suggest that there has been a need for cuckoos to advance their breeding arrival time. In the UK, however, the cuckoo has not advanced its spring arrival in line with other species [21]. While the cuckoo has undergone substantial recent population decline in the UK, particularly England [22], host availability is unlikely to have driven this [23]. This is because large declines in English cuckoos occurred where reed warbler (*Acrocephalus scirpaceus*), a late-breeding long-distance migrant that is increasing in availability, is being used more frequently [9]. Increased parasitism of reed warblers by cuckoos in England [23,24] could therefore be at least partly due to constraints on adjustment of its arrival to the breeding area. While this shift in host use reduces further requirement for advance in arrival, any relationship to the species' population decline would have to be indirect.

This study aims to understand why there has been limited advance in timing of breeding grounds arrival of cuckoos breeding in the UK. We use 11 years of satellite tracking data from 87 male cuckoos tagged at 11 sites to examine variation in migratory timing throughout the annual cycle and its potential fitness consequences. Near real-time lifetime tracking of individuals [25] allows us to estimate their timing throughout the annual cycle in an unbiased fashion and relate this to mortality events. By focussing on males, we exclude sex as a potential source of timing variation and focus on the part of the population that is almost exclusively responsible for estimated arrival trends, due to the high detectability of the male's song. While females are directly responsible for the selection of hosts, there is evidence that male as well as female cuckoos specialize on parasitizing specific host species [22,23].

We undertake three main investigations to identify where potential timing constraints may be operating and whether there is evidence that the observed migratory timing is the result of trade-offs between survival and reproduction.

First, (a) we examine variation in, and repeatability of, migratory timing across the annual cycle to assess potential constraints on response to environmental change. The degree of timing variation across the population indicates the width of the optimal window for migration and hence likelihood of timing constraints, whether due directly to environmental conditions or to scheduling of later events. Repeatability is the proportion of timing variance accounted for by consistent differences between individuals, with low repeatability indicating a role for environmental factors and high repeatability indicating a role for endogenous control or other sources of consistent individual differences. An event that is environmentally constrained should show high population synchrony and low repeatability. High repeatability but also high synchrony would suggest endogenous constraints, such as arising through non-shifting timing cues.

Second, (b) we examine how arrival at the breeding grounds is impacted by timing of previous migration stages. Using path analysis, we quantify both the direct and indirect effect of each migration event (and those variables impacting it) on breeding grounds arrival. The cumulative strength of these direct and indirect pathways indicates an event's potential to operate as a constraint. Changes in habitat quality influencing the abundance of key prey species (primarily large Lepidopteran larvae) for adult cuckoos [24] have been implicated as causes of regional UK cuckoo population declines. Elevated mortality during use of a recently discovered southwestern post-breeding migration route via Spain, compared with during migration via Italy, is contributing to the decline over and above, but possibly in interaction with, effects of local habitat change [26]. Hence we assess and control for the impact of major breeding habitat and post-breeding migration route in this analysis. We also include breeding location co-ordinates to assess the impact of potential differences in breeding grounds phenology and, to assess the impact of migratory decisions made along the migratory route, we include the location of final pre-Sahara crossing stopover for both southbound and northbound migrations.

Third, (c) we use path analyses to assess whether timing of migration impacts mortality, and whether this varies by breeding habitat or post-breeding migration routes. Due to different selective pressures on post-breeding and pre-breeding migration [27], different patterns are predicted. Increased mortality of early migrating birds may occur after an event that is under selection for early migration due to its impact on breeding grounds arrival, as they may advance timing in a trade-off between survival and reproduction. Such trade-offs may be exacerbated by stress brought about by the need to advance breeding grounds arrival. Conversely, energetic limitation due to poor body condition may result in higher mortality in later migrating birds [8], particularly during stages of post-breeding migration that are energy, rather than time, selected [27].

By examining the role of migration stages across the annual cycle in the limited advance of cuckoo arrival to the breeding grounds, we hope to improve understanding of why some species show limited response to global change. This should allow better prediction of future responses and increased understanding of how conservation measures can potentially off-set the fitness consequences of timing stresses.

## 2. Methods

### (a) Tagging and data preparation

A total of 97 male cuckoos was tagged in the breeding seasons of 2011 to 2022 in the UK, using 5 g PTT-100 tags from Microwave Telemetry. The work was carried out under license from the Special Marks Technical Panel operating on behalf of the British Trust for Ornithology and the UK Government's Home Office. Each tag was programmed for a transmission duty cycle of 10 h on and 48 h off to allow the battery to charge via the solar panel. With four exceptions, tags deployed from 2013 were additionally programmed to transmit whenever the battery was close to fully charged but otherwise adhered to the scheduled duty cycle, providing extra positions when weather conditions were favourable. See Hewson *et al.* [26] for further details of tagging and data filtering methods.

### (b) Defining stopovers

Data from the beginning of the project until December 2022 were downloaded from Movebank. Fixes were defined as coming from different transmission cycles if they were more than 10 h apart. Stopovers were defined as periods when best locations [26] from two or more consecutive transmission periods were within 50 km of each other, but those less than one day in length were omitted. Tags often showed gradually depleting battery charge during a stopover but usually charged and began transmitting upon exposure to sunlight when a bird moved to a new location [26]. Therefore, when no locations were received for one or more transmission cycles before a bird was detected at a new location, the bird was assumed to have remained at the previous stopover location until the day the last missed location was expected.

### (c) Defining migratory milestones

**Figure 1.**
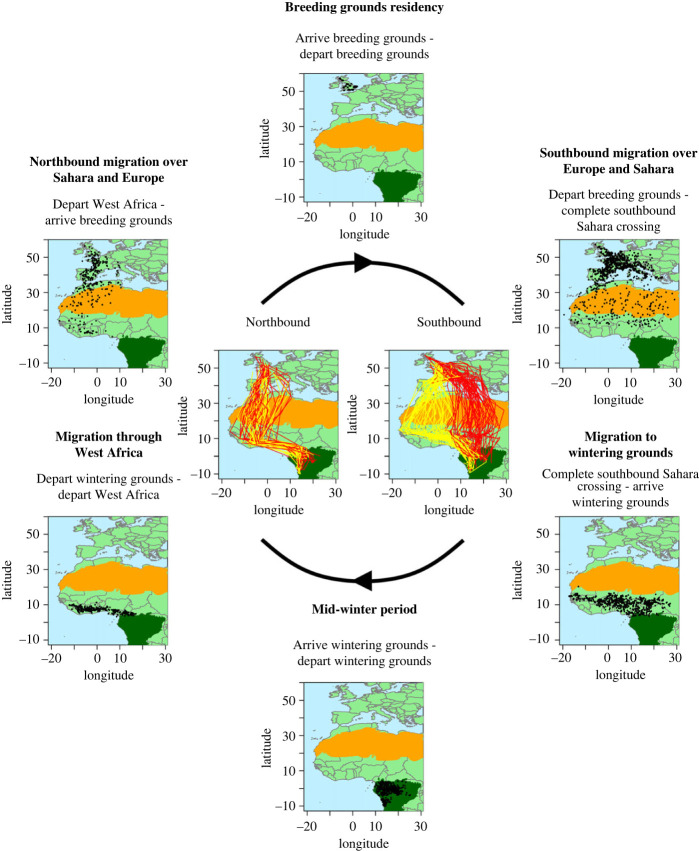
Distribution of cuckoo locations during the migratory stages delimited by the milestones defined (outer panes) and individual tracks (centre panes), showing the geographical spread of fixes in each migration stage and the use of two distinct migration routes on southbound but not northbound migrations. Shown are all locations between milestones (black points), extent of Sahara (orange polygon) and of wintering grounds (dark green polygon), and tracks of birds using the southwestern (yellow) and southeastern (red) routes during post-breeding migration.

Cuckoos exhibit a staged migratory cycle, consisting of stopovers or periods of slow movement interspersed with longer migratory movements. Six migratory milestones were defined to summarize this, designed for accuracy and reliability while representing biologically important events, such as the start or end of migratory periods or key stages within those periods. Milestones were: (1) departure from the breeding grounds; (2) completion of the Sahara crossing; (3) arrival to and (4) departure from the wintering grounds; (5) departure from the West African stopover; and (6) arrival at the breeding grounds. See figure 1 for the distribution of locations between each pair of milestones and electronic supplementary material, Methods 1 and 2 and table S1 for definitions and further details.

To minimize potential timing error, milestones whose uncertainty exceeded a certain threshold were excluded from analyses. Uncertainty was defined as the time difference between the fix on which the milestone was based, and the next or previous fix (depending on milestone definition). For departure from West Africa, in cases where milestone was based on fixes in the Sahara, uncertainty was set at two days due to the short time spent on the desert crossing. Defined thus, uncertainty was greatest for departure from the wintering grounds (mean 11.5 days). Milestones with an uncertainty above five days were then excluded, after which 604 milestones from 137 migratory cycles for 87 birds were available for analysis (electronic supplementary material, table S1).

Each tagging location was classified as ‘upland’ (Skye & Lochalsh, the Trossachs, the Forest of Bowland, the North York Moors, Wales or Dartmoor) or ‘lowland’ (Sherwood Forest, Norfolk Broads, Thetford Forest, Worcestershire, the South Downs or the New Forest), using the criteria outlined in Hewson *et al.* [26] (electronic supplementary material, figure S1). Birds were classified as using the southwestern or southeastern migratory route if they transited Iberia or Italy respectively or if the track linking successive best of day locations passed over Iberia or Italy on southbound migration. Birds lost to tracking before migratory route could be determined were assumed to be on the same migratory route as for the previous year where data existed. The migratory routes of three birds were not classifiable under the above criteria and were omitted from analyses including that variable. Birds were omitted from the dataset if they died before leaving the UK.

#### (i) Data processing

Two datasets were used: the complete set of milestones (*full dataset*); and a dataset containing only those milestones that were repeated in more than 1 year within a bird (*repeat dataset*), comprising milestones from 31 individuals after removal of uncertain milestones, of which 21 contributed from two annual cycles; 6 from three cycles; 3 from five cycles; and 1 bird contributed from six annual cycles. For the full dataset, *date* (Julian day of milestone) was used to assess timing variation between individuals. For the repeat dataset, *within-individual anomaly* (the difference between the date of the migratory milestone and the mean date of that migratory milestone for that bird across all years) was used in analyses indicated below.

### (d) Analyses

#### (i) Variation and repeatability of migratory timing

Variation in migratory timing for each milestone was summarized using variance. The change in variance between consecutive milestones was assessed using *F*-tests. Within- and between-individual variation in migratory timing were estimated using variance components models (i.e. no fixed effects), with date as the response variable for a given milestone and individual as a random effect. The variance of the random effect for individual and the residual variance represent the between- and within-individual variance respectively. Repeatability (a measure of consistent individual differences) is the proportion of total variance attributable to between-individuals.

Mixed models were fitted in a Bayesian paradigm using Stan [28], accessed through the R package ‘brms’ [29]. For models fitted in Stan, the first 2000 iterations of the MCMC chains were discarded as burn-in, and the next 4000 iterations were retained for estimating the posterior probability distributions for the parameters. Models were considered to have converged if the Gelman-Rubin statistic Rhat was less than 1.1 for all parameters.

#### (ii) Path analysis—breeding grounds arrival time

Relationships between the timing of all milestones and their contribution to determination of the timing of breeding grounds arrival were determined using path analysis, a type of structural equation model [30]. This allows robust estimation of the direct and indirect effects that variables have on each other, while controlling for the effects of covariates influencing timing. This model, hereafter referred to as the ‘timing model’, included the six previously defined migratory milestones as dependent variables. To control for and assess the influence of individual-level ecological characteristics and geographical variables potentially impact timing, the model also contained six independent variables: (1) breeding habitat; (2) migratory direction; (3) breeding longitude; (4) breeding latitude; (5) latitude of the last European pre-Sahara crossing stopover; and (6) longitude of the last West African pre-Saharan stopover.

Paths were initially constructed using a conceptual framework based on knowledge of the migratory system and preliminary univariate investigation using the ‘brms’ package [31]. Breeding habitat and migration route were linked to all milestones to account for scheduling differences of different migratory populations. Breeding grounds coordinates were linked to arrival to and departure from the breeding grounds to account for geographical variation in breeding phenology. The locations of the final pre-Saharan European and West African stopovers were linked to completion of the southbound Saharan crossing and departure from West Africa, respectively, to assess the effect of stopover decisions along the migratory route. All migratory milestones were linked to the next milestone and to arrival to the breeding grounds, to allow both direct and indirect carry-overs to be estimated. Additionally, arrival to the wintering grounds was initially directly linked to departure from West Africa, to account for weak carry-over to and from departure from the wintering grounds. Following tests of directed separation in the ‘piecewiseSEM’ package [31], which implements structural equation modelling via a frequentist approach, this link was replaced with a direct pathway identified between the southbound Sahara crossing and departure from West Africa. Separate models using the same structure were run with milestone date and within-individual anomalies. See electronic supplementary material, figure S2*a* for the structure for the resulting timing model & electronic supplementary material, Methods 3 for full model notation.

#### (iii) Path analysis—effects of timing on mortality

Path analysis was conducted to assess the relationship between milestone date and mortality before the next milestone, to determine the effect of timing in one migration stage on mortality risk in the next. We assessed how this effect varies with breeding habitat and migratory direction, while controlling for the direct effect of these variables on both timing and mortality rate (electronic supplementary material, figure S2*b*). This model, hereafter referred to as the ‘mortality model’, included two dependent variables: (1) timing; and (2) mortality, modelled as a binary variable, where 0 indicates survival and 1 indicates a mortality event after a specific milestone. Insufficient data prevented the application of this model to within-individual anomalies. Analysis was carried out for each milestone separately, excluding departure from the wintering grounds, as only one mortality event with adequate certainty was recorded in the subsequent migration stage. Additionally, we examined the combined effect for the three milestones following migration stages that most positively impacted breeding grounds arrival (southbound Sahara crossing, departure from West Africa, and arrival to the breeding grounds). See electronic supplementary material, Methods 3 for further details and electronic supplementary material, Methods 4 for details of exclusion of potential tag failure events.

Due to relatively restricted sample sizes, and issues of missing data, we estimated the parameters of the path analyses using Markov chain Monte Carlo (MCMC) simulations within a Bayesian framework, which is robust with low sample sizes [30], and also allows for imputation of missing observations in the predictor variables while accounting for uncertainty in the imputation process. The models were implemented in JAGS 4.3.1 [32] called from R with the package ‘R2jags’ [33]. The path coefficients obtained from these analyses are equivalent to regression coefficient. The percentage of variance (R2) explained by paths in the timing model can be calculated by squaring standardized path coefficients; indirect effects are the product of each step in the path to the independent variable. See electronic supplementary material, Methods 3 for further details.

## 3. Results

### (a) Variation and repeatability of migratory timing

**Figure 2.**
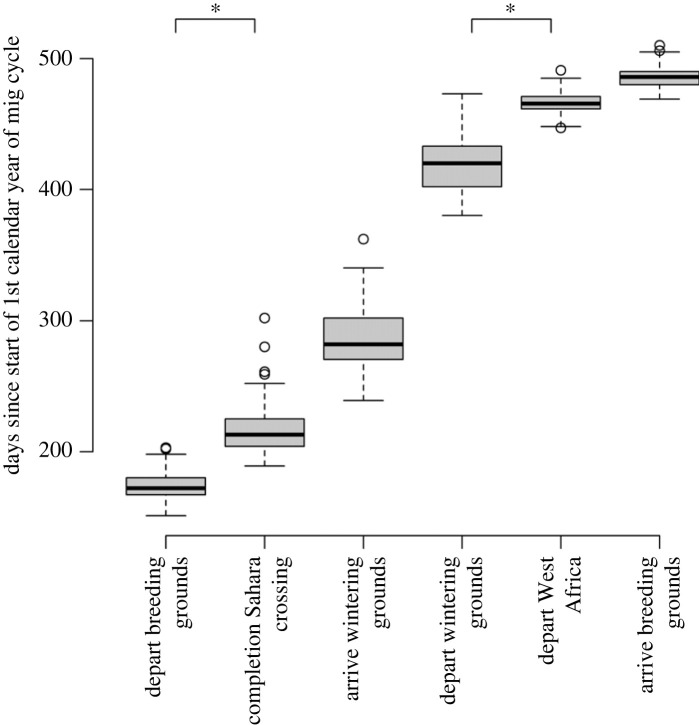
Variation in timing for each milestone, showing greater variation away from the breeding grounds and a minimum at departure from West Africa. Bold black lines show the median value; the box contains the upper and lower quartiles; the whiskers extend to 1.5 × Inter Quartile Range of the upper/lower quartiles; other data points displayed as points. Asterisks respresent pairwise comparison of variance significantly different *p* < 0.05.

The degree of variation in migratory timing differed significantly across the annual cycle (figure 2). It was largest for movements to and from the wintering grounds within tropical Africa (maximum at arrival at wintering grounds: estimated variance 605.65, 95% CI 433.61, 844.62), then decreased sharply to its minimum at departure from the West African stopover (estimated variance 78.01, 95% CI 52.35, 116.72). It then remained low through to arrival at the breeding grounds, before increasing again during southbound migration through Europe and across the Sahara (figure 2). This suggests that specific timing is more important, or more constrained, closer to the breeding grounds, especially during the pre-breeding migration and particularly at departure from the West African stopover.

Repeatability of timing was low to moderate for all milestones (table 1; mean 0.35, range 0.17–0.51), lowest for departure from the wintering grounds and highest at arrival to the wintering grounds (table 1). This demonstrates a stronger potential role for environmental influences than for consistent individual differences in determining timing throughout the annual cycle.

**Table 1.** Estimated repeatability for each milestone; all values are low to moderate, suggesting considerable scope for environmental influences on timing. Bold = non-overlap of 95% CI with zero. See electronic supplementary material, table S2 for within- and between-individual variance components and timing uncertainty estimates for each milestone.

milestone	Est	LCI	UCI
depart breeding grounds	**0**.**41**	**0**.**15**	**0**.**63**
completion Sahara crossing	**0**.**25**	**0**.**01**	**0**.**50**
arrive wintering grounds	**0**.**51**	**0**.**24**	**0**.**71**
depart wintering grounds	0.17	0.00	0.58
depart West Africa	**0**.**40**	**0**.**03**	**0**.**70**
arrive breeding grounds	**0**.**34**	**0**.**01**	**0**.**68**

### (b) Path analysis—determinants of breeding grounds arrival

**Figure 3.**
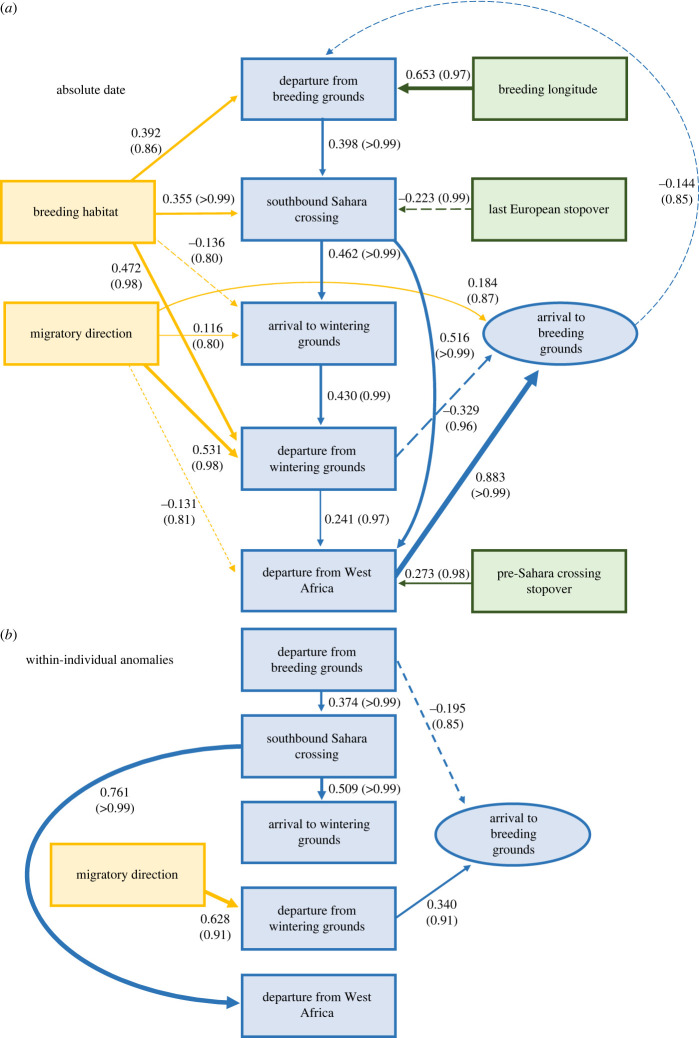
Causal relationships between the timing of migration stages and determination of breeding grounds arrival, showing the dominant impact of departure from West Africa on absolute date of arrival, but not on within-individual timing anomaly. Shown are migratory milestones (in blue), measured as (*a*) absolute dates and (*b*) within-individual anomalies, together with effects of individual/environmental (yellow) and geographical (green) variables. The most likely paths, with ≥80% probability of being non-zero, are shown; those with probability ≥0.90 are considered well-supported and those with probability ≥0.95 as very well-supported. For further details see electronic supplementary material, table S3. Breeding habitat describes the effect of breeding in the uplands relative to the lowlands. Migratory direction describes the effect of migrating in a southwest direction relative to a southeast direction. Arrows indicate direction and strength of relationships, with widths proportional to the effect strength. Solid lines indicate positive relationships, dashed lines indicate negative relationships. Estimates of standardized path coefficients and their probabilities of being non-zero (in brackets) are indicated next to the corresponding arrows.

Figure 3 shows all paths for which the coefficient differed from zero with a certainty of at least *p* = 0.8. Additional information on coefficients of all paths can be found in electronic supplementary material, table S3.

#### (i) Absolute timing

All relationships identified between milestones were very well-supported. The major determinant of timing of arrival to the breeding grounds was departure from West Africa, which accounted for 78.0% of its variation (determined by squaring the path coefficient of 0.883) (figure 3*a*). The only other direct impact on breeding grounds arrival was a small negative effect of departure from the wintering grounds (10.8%), leaving 11.2% determined by the northward journey over the Sahara and especially, given the short duration of the former, Europe. Hence, birds that left West Africa earliest—and, to lesser extents, migrated fastest from there and left the wintering grounds latest—arrived back on the breeding grounds earliest.

Indirectly, other milestones impacted breeding grounds arrival and comprised some of the effects described for the direct pathways above. The timing of departure from West Africa was influenced by carry-overs from earlier milestones, especially by the direct effect of completion of the southbound Sahara crossing, which accounted for 26.6% of its variation and therefore 20.8% of the variation in breeding grounds arrival time. Completion of the southbound Sahara crossing was itself impacted by departure from the breeding grounds, which accounted for 15.8% of its variation and thus 3.3% of breeding grounds arrival timing via this key pathway. Carry-overs through successive milestones from departure from the breeding grounds were small and thus accounted for a negligible amount of breeding grounds arrival variation, especially when accounting for the direct negative effect of departure from the wintering grounds.

There were small but well-supported effects of the location of the final pre-Saharan stopover on both southward and northward migration. Birds whose final European pre-Saharan stopover was further north were earlier, accounting for 5.0% of variation in completion of the southbound Sahara crossing and 1.0% of breeding grounds arrival. Birds whose last pre-Saharan stopover in West Africa was further east were later, accounting for 7.5% of variation in departure from West Africa and 5.8% of breeding grounds arrival.

Breeding habitat and migration route each weakly impacted four milestones and thus had very small indirect effects on breeding grounds arrival, the best supported of these effects being that lowland birds were earlier completing the southbound Sahara crossing and departing from the wintering grounds than upland birds. Also, birds migrating southwest during post-breeding migration were later departing from the wintering grounds than birds migrating southeast. Breeding longitude had a strong and very well-supported positive effect on departure from the breeding grounds (birds breeding further east departed later) which accounted for 42.6% of variation.

#### (ii) Within-individual timing anomaly

There were fewer well-supported pathways identified for within-individual anomalies, which might in part reflect the reduced power of this model to detect weaker pathways due to smaller sample sizes. Only one well-supported direct impact on breeding grounds arrival anomaly was detected (figure 3*b*), a positive effect of departure from the wintering grounds that accounted for 11.6% of variation. These results indicate that the major determinant of breeding grounds arrival anomaly was the final step of migration after departure from West Africa, accounting for 84.7–88.4% of total variation depending on whether a negative effect of departure from the breeding grounds which had some support (accounting for 3.7% of variation) is included. There were no indirect effects of milestones on breeding grounds arrival as neither departure from breeding grounds nor wintering grounds were impacted by other milestones.

### (c) Path analysis—influence of timing on mortality

Of the five milestones for which there was sufficient data after excluding uncertain mortality events, the best-supported relationships between timing and mortality occurred earlier in the annual cycle, where sample sizes were greatest (electronic supplementary material, table S4). A very well-supported effect showed that birds that left the breeding grounds later were more likely to die during the next migration stage (timing coefficient mean 2.020 95% Cis −0.199–5.659 *p* = 0.960); there was some support for an interaction with breeding habitat (breeding habitat × timing coefficient −4.181 95% Cis −12.49–3.043, *p* = 0.866), suggesting that this was more the case for lowland breeders, within which there was a very well-supported effect (lowlands coefficient 2.52, 95% Cis 0.049–5.343, *p* = 0.976), than upland breeders.

The three milestones following migration stages that most positively and directly impacted breeding grounds arrival (completion of southbound Sahara crossing, departure from West Africa and arrival at breeding grounds) all showed increased likelihood of mortality for earlier birds, but sample sizes were small and support was correspondingly weak (electronic supplementary material, table S4). After combining these three milestones, however, there was strong support for this effect (timing coefficient −1.532, Cis −3.623–0.061, *p* = 0.969), evidence that earlier birds were more likely to die following migration stages that positively impacted breeding grounds arrival.

## 4. Discussion

Departure from West Africa for the northward crossing of the Sahara was by far the most important determinant of timing of arrival to the breeding grounds. This contrasts with previous studies, which suggest that initiation of pre-breeding migration at departure from the wintering grounds is generally the most important determinant of arrival to the breeding grounds [34,35]. Across species, departure from the wintering grounds also generally has higher repeatability of migratory timing than others across the annual cycle [36], suggesting a higher level of endogenous control, so advancement at this point potentially provides a mechanism via which birds could adapt to changing resource phenology on the breeding grounds [17]. We found, however, that repeatability for cuckoos was lowest at departure from the wintering grounds and only low to moderate at departure from West Africa, suggesting significant environmental influences limit the ability of cuckoos to respond to the need for earlier arrival via evolutionary adjustment of internal programmes. The high level of population synchrony observed in cuckoos at departure from West Africa further suggests a relatively narrow optimal window of departure from this stopover and limited flexibility to adjust the timing of this event, especially as its timing was partly determined by carry over from the post-breeding migration. Furthermore, we found early cuckoos were more likely to die after migration stages that positively impact arrival to the breeding grounds, suggesting that environmentally mediated survival costs limit their ability to return earlier. Together, these results strongly suggest that the arrival of UK cuckoos at their breeding grounds is limited by the environmental conditions constraining the timing of their departure from the pre-Sahara crossing stopover in West Africa. Previous evidence of a major role for departure from a stopover site *en route* on breeding grounds arrival is limited, although extreme conditions at a pre-barrier crossing stopover constrained breeding grounds arrival of two species in a single year [37]. Our results suggest that environmental seasonality at stopovers may be more important than annual cycle stage in determining their impact on migration schedules and their flexibility. Similar results may be found in other species that, like the cuckoo, are particularly long-distance migrants with complex annual cycles [26,38], as the greater number of stopovers increases the potential for *en route* conditions to potentially constrain breeding grounds arrival [39].

### (a) Determinants of breeding grounds arrival

Limitation of the timing of departure from West Africa across the eleven years of this study is likely due to a consistent seasonal environmental influence. Conditions within Africa improve from south to north in the northern spring with the arrival of rains associated with the Intertropical Convergence Zone and continue to improve after UK cuckoos depart northwards over the Sahara (see Thorup *et. al.* [10] for a later migrating population using the same stopover). The timing of increases in resource availability across the dry-to-wet season transition, which limit fattening rates [40], probably delay individuals reaching the appropriate body condition to initiate a northward crossing of the Sahara, forming a seasonal restraint. Both the limited carry-over of timing between departure from the wintering grounds and departure from West Africa and the direct negative effect of departure from wintering grounds on arrival to the breeding grounds support this, suggesting little advantage to early departure from the wintering grounds and even some advantage to doing so later. By contrast, between-year variations within-individuals in the relative timing of arrival at the breeding location was mostly determined by subsequent conditions during northward migration across the Sahara, North Africa and Europe. This may be partly because conditions in Europe, especially temperature, are more stochastic and unpredictable, necessitating behaviours such as modifications to the migration schedule [41], as well as reducing food availability [16] and slowing accumulation of fat necessary for onward migration.

Alternatively, departure from West Africa could be inhibited by adverse winds [42], although Akesson *et al.* [43] found that wind conditions were favourable for onward migration throughout the stopovers of northern European populations of common swifts (*Apus apus*) migrating via the western part of West Africa in April and May, while populations of other species are known to vary in timing of the Sahara crossing by many months [44]. The UK population of common swift also stops off in West Africa and, like the cuckoo, has shown limited advance in spring arrival to the UK [21] and substantial recent population decline [45]. Given the strong determination of breeding grounds arrival time found in pied flycatchers (*Ficedula hypoleuca*) departing from wintering sites, rather than stopovers, in a very similar part of West Africa [46], conditions here might act as a phenological and potentially demographic bottleneck for long-distance migrants in the northern spring, irrespective of the annual cycle stage at which they are present.

The higher level of population synchrony found for cuckoos at departure from West Africa contrasts with the wintering pied flycatchers [46], which exhibited no higher synchrony than any other annual cycle stage, despite an even higher level of breeding grounds arrival dependency on this event, albeit in data from a single year only. Their longer residency period in West Africa may allow greater but individually varying potential to advance fat accumulation for earlier departure [18,47], resulting in greater individual variation in timing constraint, or their departure may be the endogenously controlled outcome of individually varying trade-offs.

In several studies, the mid-winter period has been found to reset timing differences with no evidence of carry-over effects from the post-breeding into pre-breeding migration [48] but Briedis *et al.* [35] found an effect in males only across multiple species combined, suggesting that limited power in previous studies had prevented the identification of weak sex-specific effects. For our male cuckoos, carry-over from post-breeding to pre-breeding migration is well-supported. Specifically, completion of the southbound Sahara crossing impacted the timing of departure from West Africa directly, and thus indirectly arrival to the breeding grounds, and was itself impacted by the timing of the previous departure from the breeding grounds. Hence, whatever the proximate cause of timing differences during the post-breeding migration, there appear to be important advantages to early arrival south of the Sahara. Early birds may either be better able exploit the seasonal resource peak in the Sahel that occurs soon after the main period of arrival than those arriving later, due to competitive effects or longer exposure to good conditions before they begin deteriorate [49], and/or be able to escape deteriorating late summer drought conditions in Europe. Resulting better body condition later in the annual cycle could partially off-set seasonal timing constraints in West Africa. Such effects may be important in maximizing response to timing advancement required by global change where it is constrained by seasonal factors.

### (b) Timing and mortality

The strongest evidence for a relationship between migratory timing and mortality was during southbound migration through Europe and across the Sahara. During this stage, birds that left the breeding grounds later were more likely to die, especially those from the lowlands. This could be due to energy limitation caused by greater habitat degradation and declines of caterpillar prey species in lowland habitats, where cuckoo declines have been greatest [24], potentially combined with summer droughts having a greater impact on birds that migrate from Europe later. Additionally, cuckoos parasitizing Reed Warblers (which are restricted to the lowlands) could be trading off late-season breeding opportunities against future survival [26].

Later in the annual cycle, sample sizes decrease but we found evidence that birds that migrated early during migration stages that positively impacted breeding grounds arrival were more likely to die during the next annual cycle stage (i.e. after completion of southbound Sahara crossing, departure from West Africa and arrival at the breeding grounds). The fact that some birds are migrating before the survival optimum implies there is strong selection for early breeding grounds arrival, presumably via breeding opportunities. Timing carry overs may be more likely to result in such costly trade-offs under timing stress resulting from the demands of shifting optimal breeding grounds arrival times under climate change [14]. While a strong seasonal environmental timing constraint might shift breeding arrival closer to the survival optimum by delaying early birds, reducing exposure to subsequent seasonal stochastic risks, realised migration phenology results from an interplay between endogenous and exogenous factors [48,50]. Hence, behavioural trade-offs may result in birds migrating in suboptimal condition under environmental timing constraints, putting them at increased risk of subsequent mortality [14]. Furthermore, our results include evidence of an effect during post-breeding migration, where, unlike at or close to the breeding site in spring, stochastic seasonal risks and time selection in migratory decisions are not predicted, although seasonal risks associated with late onset of rains south of the Sahara could potentially exist. This suggests that risks associated with migrating in suboptimal body condition may be traded off against potential future improved energetic condition to subsequently advance breeding arrival.

### (c) Flexibility and adjusting to global change

Our results suggests that, overall, limited scope for adjusting timing in response to global change currently exists for cuckoos breeding in the UK. Nonetheless, adjustments to the usually clearly defined and fixed schedule of stopovers during southward [26,38,48] migration and during northward migration results in some timing advancement, as shown by impacts of the locations of pre-Sahara crossing stopovers in both Europe and West Africa on breeding arrival. Such behaviour suggests that adjustments of the annual cycle schedule to the demands of climate change could be facilitated through increasing habitat quality, and therefore fattening rates [14,18], at stopovers. This could off-set seasonal constraints by both advancing timing and reducing mortality costs associated with timing-mediated trade-offs [14].

Our results help to identify the annual cycle stages at which this is most critical, thus refining suggestions for the restoration of flyway habitats [51]. In accordance with previous suggestion based on cross-species productivity patterns [52], they indicate that the greatest impact may be at the breeding grounds. Providing caterpillar-rich feeding grounds in areas close to where cuckoo hosts breed may facilitate survival of early arriving birds and reduce energetic stress during breeding and pre-migratory periods. Areas used in Europe during southbound and northbound migrations and in northern tropical Africa to recover from and prepare for each Sahara crossing are also implicated as priority areas. Understanding the dynamics of resource use at, and departure decisions from, stopovers will provide further insight into the role of habitat quality and the potential for habitat interventions to promote successful adjustments of migration schedules under global change.

## 5. Conclusion

In contrast to previous studies, we found that departure from a stopover in tropical Africa is the main determinant of breeding grounds arrival time in the cuckoo. Latitudinal variation in seasonal productivity patterns results in little scope for advancing departure from the wintering grounds as a mechanism to advance breeding arrival, as has previously been suggested as a possible response [18,34]. Hence, ecological seasonality of sites used across the annual cycle may be more important than their position in the annual cycle in determining the relationship of events to breeding grounds arrival. We find evidence that timing is under selection for early breeding grounds arrival and that individuals trade off survival to advance breeding grounds arrival during parts of both pre-breeding and, surprisingly, post-breeding migration. Additionally, we find evidence that energetic limitation at the end of the breeding residency period may also contribute to mortality risk in the subsequent migration stage and hence to fitness reductions. Such fitness reductions may form some of the demographic mechanisms linking breeding resource asynchrony to population declines, which are so far poorly understood, especially for land birds [3,4]. The role that seasonal changes in stopover quality play in limiting temporal responses to climate change, presumably via resource availability determining fuelling rates, highlights the critical role of stopover quality in successful migration, whether via timing [14], survival [26] or other functions [53] and thus the potential for facilitating responses to global change via creation of high-quality stopover habitat.

## Data Availability

Data can be found at: https://doi.org/doi:10.5061/dryad.sbcc2frc6 [54]. Code can be found at: https://doi.org/10.5281/zenodo.7986735 [55]. Supplementary material is available online [56].

## References

[1] Møller AP, Rubolini D, Lehikoinen E. 2008 Populations of migratory bird species that did not show a phenological response to climate change are declining. Proc. Natl Acad. Sci. USA **105**, 16 195-16 200. (10.1073/pnas.0803825105) PMC2571031 18849475

[2] Thackeray SJ et al. 2010 Trophic level asynchrony in rates of phenological change for marine, freshwater and terrestrial environments: phenological change across major environments. Glob. Change Biol. **16**, 3304-3313. (10.1111/j.1365-2486.2010.02165.x)

[3] Franks SE, Pearce-Higgins JW, Atkinson S, Bell JR, Botham MS, Brereton TM, Harrington R, Leech DI. 2018 The sensitivity of breeding songbirds to changes in seasonal timing is linked to population change but cannot be directly attributed to the effects of trophic asynchrony on productivity. Glob. Change Biol. **24**, 957-971. (10.1111/gcb.13960) 29152888

[4] Samplonius JM et al. 2021 Strengthening the evidence base for temperature-mediated phenological asynchrony and its impacts. Nat. Ecol. Evol. **5**, 155-164. (10.1038/s41559-020-01357-0) 33318690

[5] Saino N, Rubolini D, Lehikoinen E, Sokolov LV, Bonisoli-Alquati A, Ambrosini R, Boncoraglio G, Møller AP. 2009 Climate change effects on migration phenology may mismatch brood parasitic cuckoos and their hosts. Biol. Lett. **5**, 539-541. (10.1098/rsbl.2009.0312) 19443508 PMC2781939

[6] Møller AP et al. 2011 Rapid change in host use of the common cuckoo *Cuculus canorus* linked to climate change. Proc. R. Soc. B **278**, 733-738. (10.1098/rspb.2010.1592) PMC3030850 20843848

[7] Mynott J. 2009 Birdscapes: birds in our imagination and experience. Princeton, NJ: Princeton University Press.

[8] Lerche-Jørgensen M, Korner-Nievergelt F, Tøttrup AP, Willemoes M, Thorup K. 2018 Early returning long-distance migrant males do pay a survival cost. Ecol. Evol. **8**, 11 434-11 449. (10.1002/ece3.4569) PMC6303765 30598747

[9] Renfrew RB, Kim D, Perlut N, Smith J, Fox J, Marra PP. 2013 Phenological matching across hemispheres in a long-distance migratory bird. Diez J, editor. Divers. Distrib. **19**, 1008-1019. (10.1111/ddi.12080)

[10] Thorup K et al. 2017 Resource tracking within and across continents in long-distance bird migrants. Sci. Adv. **3**, e1601360. (10.1126/sciadv.1601360) 28070557 PMC5214581

[11] Harrison XA, Blount JD, Inger R, Norris DR, Bearhop S. 2011 Carry-over effects as drivers of fitness differences in animals: carry-over effects in animal populations. J. Anim. Ecol. **80**, 4-18. (10.1111/j.1365-2656.2010.01740.x) 20726924

[12] Marra PP, Cohen EB, Loss SR, Rutter JE, Tonra CM. 2015 A call for full annual cycle research in animal ecology. Biol. Lett. **11**, 20150552. (10.1098/rsbl.2015.0552) 26246337 PMC4571685

[13] Small-Lorenz SL, Culp LA, Ryder TB, Will TC, Marra PP. 2013 A blind spot in climate change vulnerability assessments. Nat. Clim. Change. **3**, 91-93. (10.1038/nclimate1810)

[14] Rakhimberdiev E et al. 2018 Fuelling conditions at staging sites can mitigate Arctic warming effects in a migratory bird. Nat. Commun. **9**, 4263. (10.1038/s41467-018-06673-5) 30323300 PMC6189115

[15] Lameris TK, Van Der Jeugd HP, Eichhorn G, Dokter AM, Bouten W, Boom MP, Litvin KE, Ens BJ, Nolet BA. 2018 Arctic geese tune migration to a warming climate but still suffer from a phenological mismatch. Curr. Biol. **28**, 2467-2473.e4. (10.1016/j.cub.2018.05.077) 30033332

[16] Shipley JR, Twining CW, Taff CC, Vitousek MN, Flack A, Winkler DW. 2020 Birds advancing lay dates with warming springs face greater risk of chick mortality. Proc. Natl Acad. Sci. USA **117**, 25 590-25 594. (10.1073/pnas.2009864117) PMC7568286 32989166

[17] Schmaljohann H, Both C. 2017 The limits of modifying migration speed to adjust to climate change. Nat. Clim. Change. **7**, 573-576. (10.1038/nclimate3336)

[18] Lindström Å, Alerstam T, Hedenström A. 2019 Faster fuelling is the key to faster migration. Nat. Clim. Change. **9**, 288-289. (10.1038/s41558-019-0443-7)

[19] Gill JA, Alves JA, Sutherland WJ, Appleton GF, Potts PM, Gunnarsson TG. 2014 Why is timing of bird migration advancing when individuals are not? Proc. R. Soc. B **281**, 20132161. (10.1098/rspb.2013.2161) PMC3843828 24225454

[20] Winkler DW et al. 2014 Cues, strategies, and outcomes: how migrating vertebrates track environmental change. Mov. Ecol. **2**, 10. (10.1186/2051-3933-2-10)

[21] Newson SE, Moran NJ, Musgrove AJ, Pearce-Higgins JW, Gillings S, Atkinson PW, Miller R, Grantham MJ, Baillie SR. 2016 Long-term changes in the migration phenology of UK breeding birds detected by large-scale citizen science recording schemes. Ibis **158**, 481-495. (10.1111/ibi.12367)

[22] Massimino D et al. 2022 BirdTrends 2022: trends in numbers, breeding success and survival for UK breeding birds. BTO Research Report 753. See https://www.bto.org/our-science/publications/birdtrends/2022.

[23] Douglas DJT, Newson SE, Leech DI, Noble DG, Robinson RA. 2010 How important are climate-induced changes in host availability for population processes in an obligate brood parasite, the European cuckoo? Oikos **119**, 1834-1840. (10.1111/j.1600-0706.2010.18388.x)

[24] Denerley C, Redpath SM, Wal R, Newson SE, Chapman JW, Wilson JD. 2019 Breeding ground correlates of the distribution and decline of the Common Cuckoo *Cuculus canorus* at two spatial scales. Ibis **161**, 346-358. (10.1111/ibi.12612)

[25] British Trust for Ornithology. 2018 Cuckoo tracking project. See https://www.bto.org/cuckoos.

[26] Hewson CM, Thorup K, Pearce-Higgins JW, Atkinson PW. 2016 Population decline is linked to migration route in the Common Cuckoo. Nat. Commun. **7**, 1-8. (10.1038/ncomms12296) PMC4960304 27433888

[27] Nilsson C, Klaassen RHG, Alerstam T. 2013 Differences in speed and duration of bird migration between Spring and Autumn. Am. Nat. **181**, 837-845. (10.1086/670335) 23669545

[28] Stan Development Team. 2021. Stan modeling language users guide and reference manual, v2.29. See https://mc-stan.org/users/documentation.

[29] Bürkner PC. 2017 brms: an R package for Bayesian multilevel models using Stan. J. Stat. Softw. **80**, 1-28. (10.18637/jss.v080.i01)

[30] Grace JB, Schoolmaster DR, Guntenspergen GR, Little AM, Mitchell BR, Miller KM, Schweiger EW. 2012 Guidelines for a graph-theoretic implementation of structural equation modeling. Ecosphere **3**, art73. (10.1890/ES12-00048.1)

[31] Lefcheck JS. 2023. piecewiseSEM: piecewise structural equation modeling in R. See https://cran.r-project.org/web/packages/piecewiseSEM/vignettes/piecewiseSEM.html. 10.1177/00238309211066086 35000483

[32] Plummer M. 2003 JAGS: a program for analysis of Bayesian graphical models using Gibbs sampling. See https://www.r-project.org/conferences/DSC-2003/Proceedings/Plummer.pdf.

[33] Su YS, Yajima M. 2021. R2jags: using R to run ‘JAGS’. See https://CRAN.R-project.org/package=R2jags.

[34] Schmaljohann H. 2019 The start of migration correlates with arrival timing, and the total speed of migration increases with migration distance in migratory songbirds: a cross-continental analysis. Mov. Ecol. **7**, 25. (10.1186/s40462-019-0169-1) 31417677 PMC6689889

[35] Briedis M et al. 2019 A full annual perspective on sex-biased migration timing in long-distance migratory birds. Proc. R. Soc. B **286**, 20182821. (10.1098/rspb.2018.2821) PMC6408886 30963841

[36] Franklin KA, Nicoll MAC, Butler SJ, Norris K, Ratcliffe N, Nakagawa S, Gill JA. 2022 Individual repeatability of avian migration phenology: a systematic review and meta-analysis. J. Anim. Ecol. **91**, 1416-1430. (10.1111/1365-2656.13697) 35385132 PMC9546039

[37] Tøttrup AP, Klaassen RHG, Kristensen MW, Strandberg R, Vardanis Y, Lindström Å, Rahbek C, Alerstam T, Thorup K. 2012 Drought in Africa caused delayed arrival of European Songbirds. Science **338**, 1307-1307. (10.1126/science.1227548) 23224549

[38] Willemoes M, Strandberg R, Klaassen RH, Tøttrup AP, Vardanis Y, Howey PW, Thorup K, Wikelski M, Alerstam T. 2014 Narrow-front loop migration in a population of the common cuckoo cuculus canorus, as revealed by satellite telemetry. PLoS ONE **9**, e83515. (10.1371/journal.pone.0083515) 24421890 PMC3885432

[39] Patchett R, Finch T, Cresswell W. 2018 Population consequences of migratory variability differ between flyways. Curr. Biol. **28**, R340-R341. (10.1016/j.cub.2018.03.018) 29689204

[40] Studds CE, Marra PP. 2011 Rainfall-induced changes in food availability modify the spring departure programme of a migratory bird. Proc. R. Soc. B **278**, 3437-3443. (10.1098/rspb.2011.0332) PMC3177634 21450737

[41] Senner NR, Verhoeven MA, Abad-Gómez JM, Gutierrez JS, Hooijmeijer JC, Kentie R, Masero JA, Tibbitts TL, Piersma T. 2015 When Siberia came to the Netherlands: the response of continental black-tailed godwits to a rare spring weather event. J. Anim. Ecol. **84**, 1164-1176. (10.1111/1365-2656.12381) 26033015

[42] Loonstra AHJ, Verhoeven MA, Senner NR, Both C, Piersma T. 2019 Adverse wind conditions during northward Sahara crossings increase the in-flight mortality of Black-tailed Godwits. Ecol. Lett. **22**, 2060-2066. (10.1111/ele.13387) 31529603 PMC6900105

[43] Åkesson S, Bianco G, Hedenström A. 2016 Negotiating an ecological barrier: crossing the Sahara in relation to winds by common swifts. Phil. Trans. R. Soc. B **371**, 20150393. (10.1098/rstb.2015.0393) 27528783 PMC4992717

[44] Verhoeven MA, Loonstra AHJ, Senner NR, Mcbride AD, Both C, Piersma T. 2019 Variation from an unknown source: large inter-individual differences in migrating black-tailed godwits. Front. Ecol. Evol. **7**, 31. (10.3389/fevo.2019.00031)

[45] Woodward ID et al. 2020 BirdTrends 2020: trends in numbers, breeding success and survival for UK breeding birds. BTO Research Report 732. See https://bto.org/our-science/publications/birdtrends/2020.

[46] Ouwehand J, Both C. 2017 African departure rather than migration speed determines variation in spring arrival in pied flycatchers. Chapman J, editor. J. Anim. Ecol. **86**, 88-97. (10.1111/1365-2656.12599) 27726147

[47] Bayly NJ, Norris DR, Taylor PD, Hobson KA, Morales-Rozo A. 2020 There's no place like home: tropical overwintering sites may have a fundamental role in shaping migratory strategies. Anim. Behav. **162**, 95-104. (10.1016/j.anbehav.2020.02.003)

[48] Åkesson S, Helm B. 2020 Endogenous programs and flexibility in bird migration. Front. Ecol. Evol. **8**, 78. (10.3389/fevo.2020.00078)

[49] Trierweiler C, Mullié WC, Drent RH, Exo K-M, Komdeur J, Bairlein F, Harouna A, De Bakker M, Koks BJ. 2013 A Palaearctic migratory raptor species tracks shifting prey availability within its wintering range in the Sahel. J. Anim. Ecol. **82**, 107-120. (10.1111/j.1365-2656.2012.02036.x) 23137184

[50] Stanley CQ, Hallager SH, Dudash MR, Marra PP. 2022 Food limitation modulates the endogenous control of spring migratory behavior in a captive long-distance migratory bird population. Behav. Ecol. Sociobiol. **76**, 136. (10.1007/s00265-022-03242-1)

[51] Vickery JA et al. In press. The conservation of Afro-Palaearctic migrants: what we are learning and what we need to know? Ibis **165**. See https://onlinelibrary.wiley.com/doi/abs/10.1111/ibi.13171.

[52] Morrison CA et al. 2021 Covariation in population trends and demography reveals targets for conservation action. Proc. R. Soc. B **288**, 20202955. (10.1098/rspb.2020.2955) PMC7934962 33653129

[53] Schmaljohann H, Eikenaar C, Sapir N. 2022 Understanding the ecological and evolutionary function of stopover in migrating birds. Biol. Rev. **97**, 1231-1252. (10.1111/brv.12839) 35137518

[54] Davies JG, Kirkland M, Miller MGR, Pearce-Higgins JW, Atkinson PW, Hewson CM. 2023 Data from: Spring arrival of the common cuckoo at breeding grounds is strongly determined by environmental conditions in tropical Africa. Dryad Digital Repository. (10.5061/dryad.sbcc2frc6) PMC10281800 37339739

[55] Davies JG, Kirkland M, Miller MGR, Pearce-Higgins JW, Atkinson PW, Hewson CM. 2023 Spring arrival of the common cuckoo at breeding grounds is strongly determined by environmental conditions in tropical Africa. Zenodo. (10.5281/zenodo.7986735) PMC10281800 37339739

[56] Davies JG, Kirkland M, Miller MGR, Pearce-Higgins JW, Atkinson PW, Hewson CM. 2023 Spring arrival of the common cuckoo at breeding grounds is strongly determined by environmental conditions in tropical Africa. Figshare. (10.6084/m9.figshare.c.6688811) PMC10281800 37339739

